# Pyæmia and Hospital Gangrene

**Published:** 1862-01

**Authors:** 

**Affiliations:** Berlin


### PYÆMIA AND HOSPITAL GANGRENE.

By Prof. JUNGKEN, of Berlin.

Reprinted from *Med. Times and Gazette.*

These diseases are more easily prevented than cured. For prevention, it is necessary that the most scrupulous cleanliness should be rigorously carried out in the treatment of the wounded, and, where many patients with ulcerations are congregated in rooms, thorough ventilation should be attended to. Where the surgeon has the choice, he should object to the reception of a large number of wounded persons in one common ward, as this is only too often the cause of pyæmia and hospital gangrene. Where there are no other contrivances for ventilation, the doors and windows should be opened, even if the patients complain of cold. The patients should also be frequently bathed. If the wounds are severe and the suppuration considerable, the perpetual bath is very useful for preventing these diseases; but the water should be often changed so that it does not become impregnated with the pus. Local baths, where they can be had, are also very useful; otherwise, a half or a whole bath ought to be given. Concerning local baths, Prof. Jungken condemns such tubs as are furnished with caoutchouc rings, through which the limbs are put, as, in order to prevent the water from flowing out of the tub, the caoutchouc ring must fit the limb so closely that the veins thereby become compressed, and thus a free circulation is impeded and mortification induced. If the bath is to be useful to the patient, the limb must rest in the water quite free and without any troublesome pressure. Fumigations with chlorine or vinegar are only feeble palliative means, which may be applied where no fresh and pure air is to be had. If fumigations must be made, those recommended by Guyton with chlorine are preferable to those with vinegar; but they never purify the air of sick-rooms so thoroughly as is done by fresh air, and in many patients they produce troublesome symptoms on the part of the respiratory organs.

As wars are generally undertaken in the warm, fine seasons, these being favorable to the movements of large armies, it is just at this season that great numbers of wounded have to be treated. The locality of great battles being dependent upon accidents which cannot be foreseen, the surgeon generally finds himself under the necessity to provide accommodation for the wounded in rooms not at all suitable for the purpose. In order, therefore, to prevent pyæmia and hospital gangrene under such circumstance, the surgeon ought to prescribe the air-bath, that is, to order the wounded to be placed in the open air under linen tents.

Delpech, the great French surgeon, did not recommend the air-bath so strongly as Brugmanns, who was the first to employ it; but he erred in employing the air-bath alone, without other remedies, for the cure of hospital gangrene. According to Professor Jungken, there is no specific remedy for this obstinate and dangerous affection, which could, exclusive of all others, effect a complete cure. Not even the actual cautery is able to do this, although it is the most powerful of all remedies offered to us for combating this affection; but even if we cauterize a gangrenous wound as deeply as possible, and the patient is still left in a contaminated atmosphere, the ulcer will again present a gangrenous aspect as soon as the eschar has come away.

The air-bath is just as important in pyæmia as it is in hospital gangrene, especially if it is possible to place the patient under trees in full leaf, the exhalations of which are known to have a beneficial effect. In many desperate cases of pyæmia and nosocomial gangrene, where every means had been employed in vain, and the patients seemed past hope, an immediate change for the better was perceived after Prof. Jungken had ordered them to be taken out of the surgical wards of the Charité Hospital, into the gardens adjoining this institution, where they were placed on a simple couch, covered with a blanket, and left in the open air for the rest of the day. Other remedies which had until then been employed in vain now took effect, and the Professor thus succeeded in saving wounded persons who must otherwise have certainly perished.

Professor Jungken found a striking confirmation of these views during the revolution in Dresden, when many a sanguinary struggle took place between the insurgents and the soldiers. Many of those wounded on this occasion were brought into the palace belonging to Count Marcolini, in which the saloons are furnished with very large windows, reaching to the bottom, and which looked upon a beautiful garden filled with splendid old lime-trees. Whenever the weather permitted, those most dangerously wounded were carried on their beds into the garden, and spent the whole day under the trees. The results were strikingly favorable. It is, after all, better that the patient should shiver a little in a cold but pure air, than that he should die in a warm but poisoned atmosphere.

It is also of great importance to prevent any intercourse between those affected with hospital gangrene and others. The bandages and instruments which have been employed for gangrenous wounds ought not, if possible, to be employed a second time, as the severity of the sufferings is thereby increased in those already affected, and others may receive the contagion in this way. Bandages, linen, or clothing, etc., should not be kept in rooms where patients so infected lie, neither should lint, etc., be prepared there. A frequent change of the bedding, blankets and linen of such patients is also of the greatest utility.

Among the large number of local remedies which have from time to time been recommended for hospital gangrene, the more effective are per-chloride of iron, the acidum pyrolignosum, chloride of lime, hydrochloric acid, carbon powder with chloride of zinc, myrrh and camphor (℞. Carb. lign. pulv. ℥j; myrrh ʒj; camph. ras. ʒss.; zinc. chlo. grs. v. in f. pulv.), and chloride of zinc in solution. Of all the other remedies little or nothing is to be expected. But the medicines just mentioned have not the same effect at all times. There are periods in which chloride of lime is particularly effective, and when no other remedial agents will answer the purpose; at other periods chloride of lime entirely fails, and carbon powder or acidum-pyro-lignosum produce good results, so that it is probable that, in spite of the similarity and even apparent identity of symptoms observed, there must be some peculiar differences in the several epidemics of hospital gangrene, which are at present only to be recognized by the success and failure of certain remedies.

There is only one remedy for this terrible disease, which never fails the surgeon, provided it is properly used, and that the other circumstances, such as light, air, etc., are favorable: this is the actual cautery, which has of late been unjustifiably neglected, but is believed by Prof. Jungken to be the only certain and reliable means for destroying the peculiar and dangerous contagion of hospital gangrene. To neglect the actual cautery under such circumstances, is not only to do harm to the infected patients, but also to those not yet attacked, as the contagion will then no doubt spread further, and become more dangerous. The surgeon ought to be the readier to employ the actual cautery, as the patient may be put under the influence of chloroform, and thus be spared much suffering during the operation. To render this effective, the iron must be brought to white incandescence, in order that the most intense heat may be applied to the gangrenous surface; otherwise the operation is useless. It must also be applied at an early period—if possible, at the commencement of the epidemic—in order to destroy the evil in the germ. If, however, the gangrene has become strongly developed, the whole surface of the wound must, previous to the cauterization, be laid bare, sinuosities and canals must be carefully divided, larger mortified parts must be removed by means of the scissors or the knife, the surface must be cleaned of all detritus, and dried by lint or sponges, so that the cautery may act upon a clean and dry surface. It must be applied especially to the edges of the ulcer, and beyond the diseased parts, in order to produce plastic re-action. Afterwards the patient must not remain in the room in which he was before; but he ought to be transferred either into the open air or in a single room, where doors and windows are open by night and day; and the bed ought to stand as close as possible to the window, even during the night, and whether the season be rough or not. The elimination of the eschars ought to be left to nature, and should not be promoted by warm fomentations or cataplasms, which relax the parts and thus favor relapses. The eschars will then come off a little later, but we have, on the other hand, the advantage of a strong re-action, a better suppuration and formation of granulations. Gentle irritants should afterwards be applied to the surface of the ulcer.

The local applications ought to be accompanied with a suitable dietetic and pharmaceutical treatment. The strength of the patient must be kept up, and no mercury be given, the bowels being kept open by mild aperients. By strictly adhering to these rules, Prof. Jungken has for a number of years succeeded in keeping the surgical wards of the Charitè Hospital, which were formerly a hot-bed of hospital gangrene, free from this terrible scourge.

